# Dietary Betaine Addition Alters Carcass Traits, Meat Quality, and Nitrogen Metabolism of Bama Mini-Pigs

**DOI:** 10.3389/fnut.2021.728477

**Published:** 2021-08-27

**Authors:** Yating Cheng, Mingtong Song, Qian Zhu, Md. Abul Kalam Azad, Qiankun Gao, Xiangfeng Kong

**Affiliations:** ^1^Hunan Provincial Key Laboratory of Animal Nutritional Physiology and Metabolic Process, CAS Key Laboratory of Agro-Ecological Processes in Subtropical Region, National Engineering Laboratory for Pollution Control and Waste Utilization in Livestock and Poultry Production, Institute of Subtropical Agriculture, Chinese Academy of Sciences, Changsha, China; ^2^College of Advanced Agricultural Sciences, University of Chinese Academy of Sciences, Beijng, China; ^3^Research Center of Mini-Pig, Huanjiang Observation and Research Station for Karst Ecosystems, Chinese Academy of Sciences, Beijing, China

**Keywords:** Bama mini-pig, betaine, carcass traits, meat quality, nitrogen metabolism

## Abstract

Betaine is widely used as feed additives in animal husbandry as it can cause many benefits such as improving antioxidant ability, growth performance, and carcass traits. However, there are limited studies about the effects of betaine on the Bama mini-pigs. The present study was conducted to evaluate the effects of dietary betaine on carcass traits, meat quality, and nitrogen metabolism of pigs. Twenty-six pregnant Bama mini-pigs and then 104 weaned piglets were assigned for experimental treatments. The plasma and muscle samples were collected at 65-, 95-, and 125-d-old pigs, respectively. The results showed that betaine addition in the sow-offspring diets increased the lean meat rate in the 65-d-old pigs, whereas carcass weight, carcass yield, and loin-eye area were increased in the 95-d-old pigs, and carcass weight and backfat thickness in the 125-d-old pigs. Dietary betaine addition in the sow-offspring diets increased the contents of plasma Asp of 65-d-old, Met of 95- and 125-d-old, and Sar of 125-d-old pigs. Moreover, betaine addition increased the contents of Met, His, Ile, and Phe in *Longissimus thoracis et lumborum*, whereas those contents were decreased in *biceps femoris* and *psoas major* muscles at different stages. Betaine addition in the sow and piglets' diets regulated the muscle fiber-type and myogenic regulatory gene expressions. In summary, betaine addition in the sow and sow-offspring diets could improve the carcass traits and meat quality by altering the plasma biochemical parameters, amino acid composition, and gene expressions of skeletal muscle.

## Introduction

Pork is the most widely-eaten meat globally, followed by poultry and beef, and the consumption is increasing continuously (1). The demand for pork with better quality, higher nutritional value, and better taste has also increased simultaneously. Therefore, increasing attention has been paid to improve the carcass traits and meat quality (2). In the recent past, antibiotics were widely used to enhance the growth rate, feed intake, and disease resistance, as well as to improve the carcass traits and meat quality (3).

Owing to the ban on in-feed antibiotics in swine production, safer and more effective feed additives with similar effects to antibiotics are indispensable to explore. Betaine (trimethylglycine) is a non-toxic amino acid derivative commonly found in most organisms (4). Furthermore, betaine is widely used as a feed additive in animal husbandry because of its benefits on improving the antioxidant ability, growth performance, and carcass traits (5, 6). Moreover, betaine can involve in the metabolisms of protein, amino acid, and fatty acid of the body (7). Besides the dietary nutrients, betaine can also regulate the growth performance and meat quality of pigs. Maternal nutrients also play a vital role in offspring's traits. During pregnancy, fetal nutrients are derived from the mammal circulation, and the nutrition deficiency of sows may cause the intrauterine growth retardation (IUGR) of pigs (8). The IUGR pigs can hardly adapt to the environment and mainly died after delivery due to the low potential of growth, immune, and digestion (9). Moreover, several studies demonstrated that increasing the maternal diet's energy can promote the development of muscles of the piglets (10, 11). Therefore, changing the maternal nutrients may affect the growth of the offspring. However, there are limited studies about maternal betaine addition on the offspring's carcass traits, meat quality, and nitrogen metabolism.

China is the leading country for swine production, has more than 100 local pig breeds due to the variation in the natural environment and differences in socio-economic conditions (12, 13). Bama mini-pig is one of the local pig breeds in China, is genetically stable and smaller in size. Due to its anatomy and physiological similarities to humans', Bama mini-pigs are a suitable pig breed for medical models, generally used to study metabolic diseases of human beings (14–16). However, Bama mini-pig is also a high-quality local pig breed with high economic value in China because of its characteristics of high tolerance of rough feed, disease resistance, and good adaptability (17). Therefore, Bama mini-pigs play a key role in poverty alleviation through industrial development at their place of origin (18). However, the small nesting range and extensive management methods have resulted in slow growth and low lean meat rate, which may greatly decrease the economic value of Bama mini-pigs and inhibit the development of practical production. Our previous studies have found that the betaine (3,500 mg kg^−1^) supplementation to sows during pregnancy and lactation could enhance the reproduction performance, plasma reproductive hormones, and colostrum composition in Bama mini-pigs (19–21). Moreover, we have also found that betaine addition in sow and offspring diets could improve the average daily gain, feed to gain (F/G), and final weight of Bama mini-piglets during the 125 days trial (unpublished results). Therefore, based on the roles of betaine in pig nutrition, we hypothesized that the betaine addition in sow and offspring diets might affect the carcass traits and meat quality by regulating the nitrogen metabolism of pigs. Thus, the present study was conducted to investigate the effects of dietary betaine addition in sow and offspring diets during pregnancy and lactation on the carcass traits, meat quality, and nitrogen metabolism of Bama mini-pigs.

## Materials and Methods

### Animals, Diets, and Feeding

Twenty-six healthy pregnant Bama mini-pigs with similar body conditions with three to seven parities were selected and randomly divided into two groups as follows: (a) control group, sows fed a basal diet (*n* = 12) and (b) betaine group, sows fed a basal diet with 3,500 mg kg^−1^ betaine hydrochloric (*n* = 14). Dietary betaine (purity; ≥ 95%) was purchased from Sunwin Biotech Shandong Co., Ltd (Shandong, China). The sows were housed individually in gestation crates (2.2 × 0.6 m) from mating to day 104 of gestation. On day 105 of gestation, the sows were transferred to individual farrowing crates (2.2 × 1.8 m) with a heated floor pad for offspring piglets with freely accessible *ad libitum* water. After weaning, at 35-d-old, a total of 104 piglets close to the average body weight of litter were selected from the two experimental groups (48 piglets from the control group and 56 piglets from the betaine group) and divided into three dietary treatments as follows: (a) control group, piglets from the control group fed a basal diet (*n* = 48); (b) sow-betaine group, piglets from the experimental group fed the basal diet (*n* = 28); (c) sow-offspring-betaine group, piglets from the betaine group fed a basal diet with 2,500 mg kg^−1^ betaine hydrochloride (*n* = 28). Dietary betaine was mixed with the basal diet, and the weaned piglets were fed by group.

During the trial period, the sows were fed pregnant sow diets from day 3 after mating to day 104 of pregnancy and fed lactating diets from day 105 of pregnancy to weaning. The piglets were fed with pre-nursery diets from 35- to 95-d-old and fed with late-nursery diets from 96- to 125-d-old. The basal diets' nutrient levels for sows and piglets met the Chinese local swine nutrient requirements (NY/T 65-2004), and the premixes met the National Research Council diet requirements (22, 23). The composition and nutrient levels of basal diets for sows are presented in Supplementary Table 1, and the composition and nutrient levels of basal diets for piglets are listed in Supplementary Table 2.

### Sample Collection

The animals were weighed on days 65, 95, and 125 after fasted 12 h, respectively. A total of 26 pigs (including 12, 7, and 7 pigs from the control group, sow-betaine group, and sow-offspring-betaine group, respectively), were selected randomly at every stage for plasma sampling and then sacrificed under commercial conditions via electrical stunning (120 V, 200 Hz) and exsanguination (24). After slaughter, *Longissimus thoracis et lumborum* (LTL), *biceps femoris* (BF), and *psoas major* (PM) muscles from the right side of each carcass were sampled. One part of LTL muscle for meat quality measurement and one part of LTL, BF, and PM muscles was stored at −20°C for chemical composition analysis (including measurement of crude protein and dry matter). After that, the remaining muscle samples were stored in sealed plastic bags at −80°C prior to quantitative analysis.

### Carcass Traits Analysis

After slaughter, the carcass was weighed after removing the head, feet, tail, and internal organs and calculated the carcass yield (dividing the carcass weight by live body weight). The backfat thickness (between sixth and seventh ribs on the left side of the carcass) were measured immediately using a caliper, and the width and thickness of cross-section of the LTL muscle at left thoracolumbar junction were measured by a caliper to calculate the loin-eye area, following the Chinese guidelines on performance measurement technology and regulations for pigs (GB8467-87, 1988).

### Meat Quality Analysis

Meat quality was evaluated by determining marbling score, muscle color, pH, shear force, and drip loss of the LTL muscle. The marbling score was scored according to the NPPC colorimetric plate. Muscle color was measured at 24 h after slaughter by a colorimeter (Minolta CR-400; Konica Minolta, Tokyo, Japan) using the CIELAB trichromatic system as lightness, redness, and yellowness (A 90° standard observer angle, 8 mm diameter aperture, and D65 standard illuminant). The pH value was measured by direct insertion of an electrode (HI9125; Hanna Instruments, Padova, Italy) at 45 min and 24 h after slaughter stored at 4°C (1 cm deep into the LTL muscle), and the meter was previously calibrated with two buffer solutions of pH 4.0 and pH 7.0 at 25°C. The drip loss was measured 4 h after slaughter as previously described (25). Briefly, the LTL muscle samples were trimmed of adjacent fat and weighed, then hung in a plastic bag, sealed, and stored at 4°C for 24 h. Then the meat was weighed again to calculate drip loss, and defined as the loss in weight after 24 h. For the shear force analyses, the samples were cooked in a boiled water until the internal temperature reached 70°C, then the cooked samples were cooled at room temperature and cut into six stripes per sample (1 cm thick). Finally, the shear force was measured by using a texture analyzer (model HOUNSFILD-H5KS, UE).

### Chemical Composition Analysis of Muscle

The muscle samples were cut into thin slices, dried in a vacuum freeze dryer at (10 ± 5) Pa and –(45 ± 5)°C for 48 h, and then ground into powder. The crude protein and dry matter contents were measured according to the GB/T 9695.15-2008 and GB5009.5-2010, respectively. Hydrolyzed amino acid contents in muscle samples were analyzed by an amino acid analyzer (L-8900; Hitachi, Japan) as previously described by Liu et al. (26).

### Plasma Free Amino Acid Contents

The plasma samples were obtained by centrifuging at 6,500 × *g* for 10 min, and 600 μL of supernatant was taken into a new centrifuge tube. Then an equal volume of 8% sulfosalicylic acid solution was mixed, and the protein was fully precipitated at 4°C. After centrifuging at 6,500 × *g* for 10 min, the supernatant was filtered through a 0.22-μm membrane. Finally, using the L-8900 amino acid analyzer we measured the contents of free amino acids, including alanine (Ala), arginine (Arg), aspartic acid (Asp), glutamate (Glu), glycine (Gly), histidine (His), isoleucine (Ile), leucine (Leu), lysine (Lys), methionine (Met), phenylalanine (Phe), proline (Pro), sarcosine (Sar), serine (Ser), threonine (Thr), tyrosine (Tyr), and valine (Val).

### Plasma Biochemical Parameters

The levels of plasma biochemical parameters, including alanine aminotransferase (ALT), albumin (ALB), alkaline phosphatase (ALP), ammonia (AMM), aspartate aminotransferase (AST), lactate dehydrogenase (LDH), total protein (TP), and urea nitrogen (UN) were analyzed using commercially available biochemical kits (Leadman Biochemistry Technology Company, Beijing, China) according to the manufacturers' instructions.

### Real-Time PCR Analysis

Total RNA was isolated from the LTL, BF, and PM muscles using TRIzol (Invitrogen, Shanghai, China). Beta-actin and the target genes are based on the GenBank database for pigs in NCBI, and the gene-specific primers were synthesized by Invitrogen Biotech Co., Ltd. (Shanghai, China; Supplementary Table 3). The reverse transcription-polymerase chain reaction (RT-PCR) assays were conducted using the SYBR Premix Ex Taq™ Kit (TaKaRa Biotechnology Co. Ltd., Dalian, China). The quality detection of RNA, the RT-PCR, and the synthesis of cDNA were used the methods described in the previous study (27). The RT-PCR conditions were as follows: initial denaturation at 95°C for 30 s, followed by 40 cycles of denaturation at 95°C for 5 s and annealing at 60°C for 30 s, finally extension at 72°C for 30 s. The relative gene expression was calculated using the 2^−ΔΔCT^ method (28).

### Statistical Analysis

The data were analyzed by using the general linear model in SPSS 22.0 software (IBM Corporation, 2014) to perform the one-way analysis of variance (ANOVA). The means of the different treatment groups were compared by Duncan tests. The individual pigs were considered the experimental unit. All data are presented as means ± SE unless otherwise indicated. Differences were considered statistically significant when *P* < 0.05.

## Results

### Carcass Traits

To evaluate the effects of dietary betaine on carcass traits, we assessed the carcass weight, carcass yield, fat yield, lean meat rate, backfat thickness, and loin-eye area of pigs on 65-, 95-, and 125-d-old, respectively (Table 1). In the 65-d-old pigs, the lean meat rate was increased (*P* < 0.05) in the sow-offspring-betaine group compared with the control group. However, there were no significant differences observed in carcass weight, carcass yield, backfat thickness, and loin-eye area of the 65-d-old pigs among the three different groups. In the 95-d-old pigs, the carcass weight, carcass yield, and loin-eye area of pigs in the sow-offspring-betaine group were increased (*P* < 0.05) compared with the pigs in the control and sow-betaine groups. In the 125-d-old pigs, dietary betaine addition to the sow and sow-offspring diets increased (*P* < 0.05) the backfat thickness and loin-eye area of pigs compared with the pigs in the control group.

**Table 1:** Effect of dietary betaine on carcass traits of Bama mini-pigs.

**Items**	**d-old**	**Control group**	**Sow-betaine group**	**Sow-offspring-betaine group**
Carcass weight (kg)	65	4.93 ± 0.16	5.35 ± 0.18	5.30 ± 0.36
	95	8.05 ± 0.63^b^	6.89 ± 0.65^b^	10.37 ± 0.68^a^
	125	13.56 ± 1.48^b^	16.45 ± 1.32^ab^	19.87 ± 1.57^a^
Carcass yield (%)	65	54.05 ± 0.77	54.89 ± 1.77	54.49 ± 3.62
	95	54.86 ± 1.03^b^	55.66 ± 4.90^b^	60.78 ± 1.24^a^
	125	56.12 ± 1.83	60.70 ± 1.58	61.21 ± 0.82
Fat yield (%)	65	5.82 ± 0.95	6.11 ± 2.57	5.68 ± 2.08
	95	7.65 ± 1.79	7.22 ± 1.84	7.60 ± 1.21
	125	17.90 ± 3.05	20.49 ± 2.54	20.64 ± 1.97
Lean meat rate (%)	65	10.23 ± 1.18^b^	11.10 ± 0.91^ab^	12.19 ± 1.74^a^
	95	10.76 ± 1.10	11.31 ± 1.53	11.95 ± 1.12
	125	22.54 ± 2.62	24.39 ± 1.83	24.53 ± 0.25
Back fat thickness (mm)	65	13.27 ± 0.59	13.49 ± 1.20	12.18 ± 1.05
	95	18.01 ± 1.42	15.89 ± 1.00	19.11 ± 1.16
	125	25.00 ± 1.41^b^	27.12 ± 1.22^ab^	30.42 ± 1.09^a^
Loin-eye area (cm^2^)	65	4.12 ± 0.14	4.20 ± 0.11	4.98 ± 0.55
	95	4.43 ± 0.34^b^	4.98 ± 0.62^b^	7.89 ± 0.92^a^
	125	6.69 ± 0.56	7.72 ± 0.56	8.95 ± 1.17

*Data are presented as means ± SE. Values in the same row without a common superscript letter are significantly different (P < 0.05)*.

*At 65-, 95-, and 125-d-old, the replicates of the control group were 12, 11, and 8; the sow-betaine group were 6, 7, and 6; and the sow-offspring-betaine group were 7, 7, and 6, respectively*.

### Meat Quality and Chemical Composition

The effects of dietary betaine addition in the sow and sow-offspring diets on meat quality and meat chemical composition are presented in Tables 2, 3. Compared with the control group, betaine addition to sow diets reduced (*P* < 0.05) the marbling score of LTL muscle of 65- and 95-d-old pigs, whereas betaine addition in the sow-offspring diets increased (*P* < 0.05) the shear force of LTL muscle of 65-d-old pigs and the meat brightness of 95-d-old pigs (Table 2). There were no significant differences in marbling score, meat color, drip loss, and shear force of LTL muscle of 125-d-old pigs among the three dietary groups (*P* > 0.05). Furthermore, the crude protein content of LTL muscle of 65-d-old pigs was increased in the sow-offspring-betaine group compared with the pigs in the sow-betaine group, whereas dry matter content of PM muscle was decreased in the sow-offspring-betaine group compared with the control group (*P* < 0.05). However, dietary betaine addition in the sow and sow-offspring diets did not affect (*P* > 0.05) the crude protein and dry matter contents of LTL, BF, and PM muscles of 95- and 125-d-old pigs (Table 3).

**Table 2:** Effect of dietary betaine on meat quality of Bama mini-pigs.

**Items**	**d-old**	**Control group**	**Sow-betaine group**	**Sow-offspring-betaine group**
Marbling score	65	1.67 ± 0.14^a^	1.00 ± 0.00^b^	1.43 ± 0.20^ab^
	95	1.73 ± 0.19^a^	1.00 ± 0.00^b^	1.29 ± 0.18^ab^
	125	1.50 ± 0.19	1.67 ± 0.21	1.67 ± 0.21
**Meat color**
	65	49.22 ± 1.00	50.60 ± 1.91	47.19 ± 0.50
L^*^	95	50.25 ± 0.75^b^	49.39 ± 1.75^b^	54.33 ± 1.33^a^
	125	51.22 ± 1.98	51.15 ± 2.36	47.86 ± 1.12
	65	19.47 ± 0.87	21.60 ± 1.63	19.43 ± 1.40
a^*^	95	18.69 ± 0.70	18.73 ± 0.39	18.85 ± 0.83
	125	18.03 ± 0.77	15.71 ± 0.70	16.42 ± 0.73
	65	6.99 ± 0.13	6.60 ± 0.14	6.34 ± 0.49
b^*^	95	6.57 ± 0.37	6.04 ± 0.19	6.22 ± 0.24
	125	6.99 ± 0.38	7.13 ± 0.71	6.19 ± 0.66
	65	6.54 ± 0.05	5.66 ± 0.68	6.18 ± 0.05
pH_45min_	95	6.45 ± 0.08	6.56 ± 0.04	6.67 ± 0.10
	125	6.43 ± 0.08	6.51 ± 0.11	6.51 ± 0.04
	65	5.53 ± 0.05	5.60 ± 0.23	5.70 ± 0.10
pH_24h_	95	5.47 ± 0.02	5.44 ± 0.01	5.54 ± 0.04
	125	5.46 ± 0.02	5.50 ± 0.02	5.51 ± 0.01
	65	5.85 ± 0.71	5.75 ± 1.13	5.33 ± 0.59
Drip loss (%)	95	3.48 ± 0.54	2.87 ± 0.60	3.12 ± 0.38
	125	4.44 ± 0.68	2.78 ± 0.51	3.11 ± 0.54
	65	62.37 ± 3.95^b^	77.83 ± 2.62^ab^	84.99 ± 9.77^a^
Shear force (*N*)	95	70.68 ± 5.32	64.81 ± 4.56	74.41 ± 6.12
	125	93.51 ± 8.42	90.11 ± 10.09	67.08 ± 5.04

*Data are presented as means ± SE. Values in the same row without a common superscript letter are significantly different (P < 0.05)*.

*At 65-, 95-, and 125-d-old, the replicates of the control group were 12, 11, and 8; the sow-betaine group were 6, 7, and 6; and the sow-offspring-betaine group were 7, 7, and 6, respectively*.

L^*^, lightness;

a^*^, redness;

b^*^, yellowness.

**Table 3:** Effect of dietary betaine on routine nutrient contents of Bama mini-pigs (fresh weight basis; %).

**d-old**	**Nutrient ingredients**	**Control group**	**Sow-betaine group**	**Sow-offspring-betaine group**
***Longissimus thoracis et lumborum*** **muscle**
65	Dry matter	29.12 ± 2.30	27.33 ± 0.84	28.04 ± 0.63
	Crude protein	23.25 ± 2.06^ab^	22.27 ± 0.99^b^	24.57 ± 0.78^a^
95	Dry matter	28.33 ± 1.94	28.21 ± 1.11	28.70 ± 2.28
	Crude protein	22.03 ± 1.69	22.80 ± 0.90	22.92 ± 1.30
125	Dry matter	27.82 ± 2.04	28.54 ± 3.92	26.00 ± 0.51
	Crude protein	22.20 ± 0.70	22.51 ± 0.66	22.57 ± 0.61
***Biceps femoris*** **muscle**
65	Dry matter	26.89 ± 0.85	25.94 ± 1.11	26.53 ± 1.01
	Crude protein	22.23 ± 1.55	21.97 ± 0.85	22.78 ± 0.98
95	Dry matter	26.74 ± 1.43	25.85 ± 0.81	26.19 ± 0.79
	Crude protein	23.12 ± 1.63	22.27 ± 0.70	22.14 ± 0.77
125	Dry matter	27.44 ± 2.90	26.94 ± 1.54	26.10 ± 1.00
	Crude protein	22.07 ± 1.14	21.26 ± 1.01	20.65 ± 2.40
***Psoas major*** **muscle**
65	Dry matter	28.22 ± 1.33^a^	26.84 ± 1.77^ab^	26.07 ± 1.25^b^
	Crude protein	22.28 ± 3.29	21.62 ± 1.39	22.60 ± 0.72
95	Dry matter	25.92 ± 2.05	25.96 ± 0.87	24.95 ± 1.30
	Crude protein	22.92 ± 2.25	22.47 ± 0.66	21.58 ± 1.32
125	Dry matter	24.80 ± 1.17	24.58 ± 0.87	23.97 ± 0.74
	Crude protein	21.18 ± 0.61	21.51 ± 0.47	20.98 ± 0.56

*Data are presented as means ± SE. Values in the same row without a common superscript letter are significantly different (P < 0.05)*.

*At 65-, 95-, and 125-d-old, the replicates of control group were 12, 11, and 8; sow-betaine group was 6, 7, and 6; and sow-offspring-betaine group was 7, 7, and 6, respectively*.

### Plasma Free Amino Acid Contents

Plasma free amino acid contents of pigs at three different stages are presented in Table 4. Dietary betaine addition in sow-offspring diets increased (*P* < 0.05) the plasma Asp content of 65-d-old pigs and the plasma Met content of 95- and 125-d-old pigs compared with the pigs in the control and sow-betaine groups. In addition, the plasma Glu content of 65-d-old pigs was increased (*P* < 0.05) in the sow-offspring-betaine group compared with the sow-betaine group. Moreover, the plasma Sar content of 125-d-old pigs was increased (*P* < 0.05) in the sow-offspring-betaine group compared with the other two groups.

**Table 4:** Effect of dietary betaine on plasma free amino acids of Bama mini-pigs (μmol L^−1^).

**Items**	**d-old**	**Control group**	**Sow-betaine group**	**Sow-offspring-betaine group**
Ala	65	418.20 ± 28.10	365.88 ± 4.03	408.44 ± 60.46
	95	340.23 ± 37.44	348.82 ± 68.59	251.02 ± 25.80
	125	284.27 ± 25.04	372.51 ± 30.33	302.42 ± 42.99
Arg	65	78.01 ± 6.84	77.68 ± 5.39	73.65 ± 13.23
	95	82.09 ± 9.84	94.02 ± 7.17	96.94 ± 6.38
	125	88.44 ± 6.68	98.39 ± 22.84	113.29 ± 11.8
Asp	65	7.63 ± 0.94^b^	9.58 ± 0.66^b^	14.84 ± 1.42^a^
	95	12.95 ± 2.14	13.89 ± 2.27	9.44 ± 0.54
	125	11.44 ± 1.42	10.52 ± 0.48	10.01 ± 0.70
Cit	65	26.82 ± 1.17	29.23 ± 1.22	29.75 ± 4.17
	95	36.31 ± 2.46	37.50 ± 2.29	39.63 ± 1.95
	125	41.61 ± 3.78	32.39 ± 2.81	35.23 ± 2.71
Glu	65	215.96 ± 21.03^ab^	178.54 ± 18.64^b^	296.66 ± 35.11^a^
	95	190.71 ± 17.09	222.11 ± 21.23	196.33 ± 19.22
	125	157.72 ± 13.03	183.79 ± 16.01	198.18 ± 14.02
Gly	65	551.72 ± 54.19	488.64 ± 88.64	364.64 ± 29.09
	95	483.85 ± 56.50	568.49 ± 60.88	625.81 ± 20.54
	125	546.26 ± 29.07	549.24 ± 72.80	690.19 ± 91.14
His	65	37.39 ± 1.80	40.06 ± 2.75	40.61 ± 3.12
	95	41.06 ± 2.37	36.42 ± 2.27	41.13 ± 2.95
	125	42.72 ± 2.42	43.69 ± 2.73	49.33 ± 4.02
Ile	65	100.93 ± 6.16	108.55 ± 9.33	110.45 ± 9.42
	95	89.86 ± 6.62	89.28 ± 2.39	100.88 ± 11.88
	125	86.29 ± 3.02	101.02 ± 8.80	133.51 ± 16.71
Leu	65	160.52 ± 9.45	167.17 ± 12.35	178.92 ± 14.11
	95	136.37 ± 9.49	137.69 ± 4.30	151.66 ± 18.55
	125	142.49 ± 4.53	144.58 ± 4.59	203.97 ± 24.05
Lys	65	141.13 ± 10.87	155.82 ± 17.66	134.42 ± 14.15
	95	120.29 ± 12.11	133.92 ± 11.65	126.11 ± 6.61
	125	129.19 ± 4.75	133.52 ± 5.85	174.85 ± 22.84
Met	65	12.75 ± 0.85	11.44 ± 0.46	10.14 ± 1.52
	95	6.03 ± 1.80^b^	11.07 ± 0.38^b^	12.56 ± 0.65^a^
	125	14.01 ± 0.90^b^	14.32 ± 1.56^b^	20.41 ± 0.19^a^
Orn	65	53.55 ± 4.68	55.48 ± 2.82	68.64 ± 5.32
	95	64.81 ± 8.56	71.09 ± 14.08	52.84 ± 3.38
	125	66.05 ± 6.37	50.73 ± 3.56	68.89 ± 16.00
Phe	65	78.14 ± 2.61	84.38 ± 9.68	84.83 ± 2.38
	95	80.88 ± 3.84	75.84 ± 5.65	83.18 ± 6.41
	125	89.31 ± 6.39	88.79 ± 6.85	91.25 ± 3.17
Pro	65	171.23 ± 9.61	178.72 ± 48.20	182.06 ± 27.55
	95	153.18 ± 13.21	160.59 ± 19.97	181.47 ± 14.77
	125	190.08 ± 14.21	208.01 ± 20.92	166.44 ± 20.78
Sar	65	8.45 ± 1.75	6.77 ± 0.87	13.09 ± 1.79
	95	4.92 ± 1.83	2.85 ± 0.55	2.52 ± 0.74
	125	2.26 ± 0.24^b^	3.07 ± 0.37^b^	5.37 ± 1.45^a^
Ser	65	79.55 ± 5.83	83.43 ± 9.57	87.97 ± 7.28
	95	73.37 ± 6.20	85.57 ± 5.90	76.41 ± 5.50
	125	79.78 ± 1.91	88.48 ± 3.36	98.55 ± 8.40
Thr	65	121.52 ± 10.11	134.77 ± 22.12	113.67 ± 10.10
	95	110.77 ± 10.26	129.64 ± 16.82	112.06 ± 5.77
	125	122.96 ± 7.01	133.82 ± 14.29	126.65 ± 14.93
Tyr	65	41.76 ± 5.09	55.40 ± 4.31	46.91 ± 0.97
	95	50.56 ± 4.41	59.97 ± 5.33	65.60 ± 6.31
	125	62.65 ± 3.92	59.71 ± 4.97	68.97 ± 6.70
Val	65	243.90 ± 14.39	243.20 ± 17.59	276.04 ± 23.11
	95	229.92 ± 14.16	214.00 ± 4.55	247.18 ± 31.97
	125	237.37 ± 9.88	267.48 ± 17.08	345.17 ± 43.46

*Data are presented as means ± SE. Values in the same row without a common superscript letter are significantly different (P < 0.05)*.

*At 65-, 95-, and 125-d-old, the replicates of the control group were 12, 11, and 8; the sow-betaine group were 6, 7, and 6; and the sow-offspring-betaine group were 7, 7, and 6, respectively*.

*Ala, alanine; Arg, arginine; Asp, aspartic acid; Glu, glutamate; Gly, glycine; His, histidine; Ile, isoleucine; Leu, leucine; Lys, lysine; Met, methionine; Phe, phenylalanine; Pro, proline; Sar, sarcosine; Ser, serine; Thr, threonine; Tyr, tyrosine; Val, valine*.

### Plasma Biochemical Parameters

The effects of dietary betaine addition in the sow and sow-offspring diets on plasma biochemical parameters at different stages are presented in Table 5. In the 65-d-old pigs, the sow-betaine group had higher TP activity than the pigs in the control group, whereas the sow-offspring-betaine group had higher AST activity than the pigs in the other two groups (*P* < 0.05). In the 95-d-old pigs, the sow-offspring-betaine group showed a lower (*P* < 0.05) AST activity than the pigs in the sow-betaine group. In addition, both sow-betaine and sow-offspring-betaine groups showed lower (*P* < 0.05) LDH activity compared with the control group. However, dietary betaine had no effects (*P* > 0.05) on the plasma biochemical parameters of 125-d-old pigs.

**Table 5:** Effect of dietary betaine on plasma biochemical parameters of Bama mini-pigs.

**Items**	**d-old**	**Control group**	**Sow-betaine group**	**Sow-offspring-betaine group**
ALB (g L^−1^)	65	40.63 ± 0.95	44.23 ± 1.40	42.33 ± 2.49
	95	41.89 ± 0.88	40.39 ± 1.35	40.26 ± 1.39
	125	45.05 ± 1.52	46.22 ± 1.30	46.10 ± 2.01
ALP (U L^−1^)	65	162.33 ± 9.59	165.17 ± 8.79	197.14 ± 20.76
	95	125.09 ± 13.56	118.43 ± 11.53	83.57 ± 17.32
	125	175.63 ± 18.27	138.33 ± 17.07	122.00 ± 11.67
ALT (U L^−1^)	65	61.33 ± 4.58	64.30 ± 3.25	62.71 ± 6.93
	95	55.88 ± 2.91	49.58 ± 4.83	50.51 ± 5.41
	125	51.77 ± 4.66	55.33 ± 7.00	65.90 ± 3.96
AMM (μmol L^−^)	65	213.48 ± 28.32	179.42 ± 26.33	182.50 ± 13.04
	95	465.63 ± 119.89	213.67 ± 9.99	217.04 ± 11.76
	125	301.85 ± 52.62	132.45 ± 33.80	214.86 ± 46.38
AST (U L^−1^)	65	61.08 ± 6.99^b^	62.00 ± 4.52^b^	93.71 ± 11.02^a^
	95	73.30 ± 5.53^ab^	91.43 ± 18.85^a^	46.86 ± 2.55^b^
	125	69.50 ± 12.02	56.67 ± 10.89	82.00 ± 24.09
LDH (U L^−1^)	65	410.92 ± 23.68	450.75 ± 25.03	521.57 ± 51.95
	95	474.55 ± 36.21^a^	372.50 ± 32.82^b^	355.43 ± 19.04^b^
	125	508.88 ± 45.90	451.00 ± 28.76	411.80 ± 35.37
TP (g L^−1^)	65	65.61 ± 1.12^b^	74.38 ± 2.71^a^	70.17 ± 3.53^ab^
	95	74.09 ± 1.60	72.59 ± 2.08	74.46 ± 2.12
	125	71.25 ± 2.08	68.98 ± 0.65	73.68 ± 2.61
UN (mmol L^−1^)	65	3.13 ± 0.22	4.13 ± 0.71	3.96 ± 0.37
	95	3.56 ± 0.28	2.66 ± 0.29	3.01 ± 0.47
	125	3.50 ± 0.47	3.37 ± 0.38	3.76 ± 0.35

*Data are presented as means ± SE. Values in the same row without a common superscript letter are significantly different (P < 0.05)*.

*At 65-, 95-, and 125-d-old, the replicates of the control group were 12, 11, and 8; the sow-betaine group were 6, 7, and 6; and the sow-offspring-betaine group were 7, 7, and 6, respectively*.

*ALB, albumin; ALP, alkaline phosphatase; ALT, alanine aminotransferase; AMM, ammonia; AST, aspartate aminotransferase; LDH, lactate dehydrogenase; TP, total protein; UN, urea nitrogen*.

### Hydrolyzed Amino Acid Contents in the Muscle

The changes of muscle amino acid contents in the LTL, BF, and PM muscles are presented in Table 6. In the LTL muscle, compared with the control group, betaine addition in the sow and sow-offspring diets decreased (*P* < 0.05) the Asp, Ile, and Lys contents, whereas betaine addition in the sow diets decreased (*P* < 0.05) the Phe, Val, and essential amino acid (EAA) contents of 65-d-old pigs. In the 95-d-old pigs, the contents of Asp and Ile in the sow-betaine and sow-offspring-betaine groups were increased, whereas the content of Pro was decreased compared with the control group (*P* < 0.05). Moreover, the contents of Glu, Gly, and Lys in the sow-betaine group and the content of Leu in the sow-offspring-betaine group were increased (*P* < 0.05) compared with the control group. In the 125-d-old pigs, the contents of His, Met, Phe, Thr, and Tyr were increased in the sow-betaine and sow-offspring-betaine groups, whereas the content of EAA was increased in the sow-offspring-betaine group compared with the control group (*P* < 0.05).

**Table 6:** Effect of dietary betaine on hydrolyzed amino acid content in muscle of Bama mini-pigs (fresh weight basis; %).

**Items**	**d-old**	**Control group**	**Sow-betaine group**	**Sow-offspring-betaine group**
***Longissimus thoracis et lumborum*** **muscle**
	65	1.20 ± 0.09	1.14 ± 0.06	1.18 ± 0.10
Ala	95	1.09 ± 0.14	1.17 ± 0.10	1.23 ± 0.14
	125	1.17 ± 0.08	1.28 ± 0.11	1.22 ± 0.06
	65	1.35 ± 0.09	1.29 ± 0.05	1.28 ± 0.09
Arg	95	1.25 ± 0.15	1.38 ± 0.08	1.27 ± 0.10
	125	1.34 ± 0.09	1.45 ± 0.29	1.33 ± 0.13
	65	1.82 ± 0.14^a^	1.62 ± 0.13^b^	1.63 ± 0.13^b^
Asp^1^	95	1.54 ± 0.16^b^	1.80 ± 0.11^a^	1.83 ± 0.15^a^
	125	1.76 ± 0.11	1.81 ± 0.14	1.82 ± 0.07
	65	2.64 ± 0.20	2.49 ± 0.11	2.61 ± 0.17
Glu^2^	95	2.62 ± 0.28^b^	2.92 ± 0.21^a^	2.66 ± 0.24^b^
	125	2.84 ± 0.23	3.03 ± 0.22	3.11 ± 0.20
	65	1.08 ± 0.10	0.93 ± 0.09	0.99 ± 0.22
Gly	95	0.94 ± 0.13^b^	1.08 ± 0.05^a^	0.93 ± 0.10^b^
	125	0.98 ± 0.09	1.03 ± 0.11	0.94 ± 0.04
	65	0.80 ± 0.08	0.80 ± 0.14	0.88 ± 0.20
His	95	0.92 ± 0.18	0.79 ± 0.08	0.80 ± 0.05
	125	0.89 ± 0.04^b^	1.07 ± 0.10^a^	1.14 ± 0.08^a^
	65	0.90 ± 0.07^a^	0.83 ± 0.04^b^	0.81 ± 0.03^b^
Ile	95	0.83 ± 0.07^b^	0.94 ± 0.08^a^	0.93 ± 0.06^a^
	125	0.99 ± 0.07	1.04 ± 0.07	1.08 ± 0.04
	65	1.75 ± 0.14^a^	1.65 ± 0.07^ab^	1.61 ± 0.08^b^
Leu	95	1.48 ± 0.12^b^	1.58 ± 0.10^b^	1.75 ± 0.13^a^
	125	1.67 ± 0.11	1.75 ± 0.13	1.77 ± 0.08
	65	1.78 ± 0.13^a^	1.64 ± 0.09^b^	1.64 ± 0.05^b^
Lys	95	1.62 ± 0.14^b^	1.83 ± 0.18^a^	1.75 ± 0.15^ab^
	125	1.89 ± 0.14	1.97 ± 0.14	1.97 ± 0.07
	65	0.53 ± 0.04	0.51 ± 0.03	0.53 ± 0.06
Met	95	0.54 ± 0.06	0.54 ± 0.04	0.54 ± 0.04
	125	0.62 ± 0.04^b^	0.68 ± 0.04^a^	0.71 ± 0.03^a^
	65	0.87 ± 0.06^a^	0.80 ± 0.05^b^	0.89 ± 0.06^a^
Phe	95	0.86 ± 0.08	0.86 ± 0.09	0.81 ± 0.06
	125	0.90 ± 0.07^c^	1.00 ± 0.06^b^	1.11 ± 0.09^a^
	65	0.94 ± 0.09	1.01 ± 0.13	0.99 ± 0.13
Pro	95	1.18 ± 0.29^a^	1.03 ± 0.14^ab^	0.82 ± 0.12^b^
	125	1.04 ± 0.15	0.98 ± 0.13	0.88 ± 0.08
	65	0.69 ± 0.05	0.65 ± 0.03	0.66 ± 0.05
Ser	95	0.72 ± 0.09	0.75 ± 0.06	0.68 ± 0.06
	125	0.75 ± 0.06	0.84 ± 0.08	0.79 ± 0.03
	65	0.94 ± 0.08	0.89 ± 0.07	0.88 ± 0.03
Thr	95	0.86 ± 0.10	0.92 ± 0.09	0.91 ± 0.07
	125	0.96 ± 0.08^b^	1.08 ± 0.07^a^	1.09 ± 0.05^a^
	65	0.71 ± 0.05	0.67 ± 0.09	0.66 ± 0.07
Tyr	95	0.58 ± 0.24	0.68 ± 0.08	0.61 ± 0.08
	125	0.75 ± 0.10^b^	0.91 ± 0.05^a^	1.01 ± 0.13^a^
	65	0.97 ± 0.07^a^	0.90 ± 0.05^b^	0.91 ± 0.05^ab^
Val	95	0.95 ± 0.09	1.02 ± 0.07	0.98 ± 0.08
	125	1.08 ± 0.08	1.13 ± 0.09	1.15 ± 0.05
	65	18.98 ± 1.39	17.83 ± 0.78	18.16 ± 0.93
TAA	95	17.97 ± 2.00	19.30 ± 1.34	18.51 ± 1.38
	125	19.64 ± 1.37	21.04 ± 1.52	21.13 ± 0.63
	65	7.75 ± 0.59^a^	7.22 ± 0.34^b^	7.27 ± 0.27^ab^
EAA	95	7.15 ± 0.58	7.69 ± 0.61	7.68 ± 0.56
	125	8.12 ± 0.58^b^	8.64 ± 0.53^ab^	8.89 ± 0.27^a^
	65	11.23 ± 0.81	10.61 ± 0.46	10.90 ± 0.74
NEAA	95	10.83 ± 1.44	11.61 ± 0.74	10.83 ± 0.86
	125	11.52 ± 0.80	12.40 ± 1.04	12.24 ± 0.47
	65	9.31 ± 0.68	8.73 ± 0.35	8.94 ± 0.66
FAA	95	8.94 ± 1.06	9.67 ± 0.64	9.06 ± 0.75
	125	9.50 ± 0.69	10.05 ± 0.72	9.84 ± 0.40
***Biceps femoris*** **muscle**
	65	1.05 ± 0.11^b^	1.15 ± 0.03^a^	1.16 ± 0.04^a^
Ala	95	1.27 ± 0.20	1.25 ± 0.13	1.09 ± 0.06
	125	1.12 ± 0.08^b^	1.19 ± 0.05^b^	1.33 ± 0.14^a^
	65	1.27 ± 0.08	1.31 ± 0.03	1.34 ± 0.05
Arg	95	1.27 ± 0.16^a^	1.29 ± 0.12^a^	1.09 ± 0.06^b^
	125	1.29 ± 0.13	1.33 ± 0.06	1.44 ± 0.13
	65	1.69 ± 0.13^b^	2.03 ± 0.08^a^	1.94 ± 0.24^a^
Asp	95	1.86 ± 0.23^a^	1.85 ± 0.17^a^	1.55 ± 0.07^b^
	125	1.70 ± 0.16^b^	1.82 ± 0.07^b^	2.06 ± 0.20^a^
	65	2.52 ± 0.24^b^	2.85 ± 0.10^a^	2.94 ± 0.15^a^
Glu	95	2.70 ± 0.32^b^	2.97 ± 0.18^a^	2.56 ± 0.15^b^
	125	2.71 ± 0.25^b^	2.95 ± 0.14^b^	3.26 ± 0.35^a^
	65	0.83 ± 0.06^b^	1.00 ± 0.04^a^	1.02 ± 0.09^a^
Gly	95	0.88 ± 0.10^a^	0.94 ± 0.05^a^	0.80 ± 0.04^b^
	125	0.85 ± 0.08^b^	0.91 ± 0.05^ab^	1.00 ± 0.11^a^
	65	0.79 ± 0.08	0.84 ± 0.04	0.87 ± 0.08
His	95	0.91 ± 0.20	0.90 ± 0.11	0.93 ± 0.03
	125	0.93 ± 0.15	0.95 ± 0.06	1.01 ± 0.08
	65	0.86 ± 0.06^b^	0.96 ± 0.04^a^	0.93 ± 0.05^a^
Ile	95	0.92 ± 0.10^a^	0.97 ± 0.08^a^	0.82 ± 0.04^b^
	125	0.95 ± 0.08^b^	0.99 ± 0.04^b^	1.10 ± 0.12^a^
	65	1.70 ± 0.13	1.76 ± 0.07	1.69 ± 0.14
Leu	95	1.78 ± 0.25^a^	1.76 ± 0.15^a^	1.52 ± 0.07^b^
	125	1.62 ± 0.12^b^	1.68 ± 0.06^b^	1.87 ± 0.18^a^
	65	1.71 ± 0.12^b^	2.01 ± 0.08^a^	1.93 ± 0.19^a^
Lys	95	1.77 ± 0.20^a^	1.75 ± 0.14^a^	1.51 ± 0.07^b^
	125	1.79 ± 0.18^b^	1.90 ± 0.09^b^	2.14 ± 0.28^a^
	65	0.51 ± 0.04	0.55 ± 0.03	0.55 ± 0.04
Met	95	0.55 ± 0.06	0.55 ± 0.03	0.51 ± 0.05
	125	0.58 ± 0.07	0.59 ± 0.03	0.68 ± 0.11
	65	0.84 ± 0.08	0.78 ± 0.02	0.82 ± 0.10
Phe	95	0.84 ± 0.11	0.86 ± 0.04	0.81 ± 0.08
	125	0.87 ± 0.10	0.90 ± 0.07	1.03 ± 0.21
	65	0.99 ± 0.08^a^	0.63 ± 0.02^c^	0.81 ± 0.26^b^
Pro	95	0.84 ± 0.22	0.90 ± 0.20	0.82 ± 0.10
	125	0.96 ± 0.23	0.96 ± 0.18	1.00 ± 0.17
	65	0.58 ± 0.08^c^	0.70 ± 0.03^b^	0.77 ± 0.04^a^
Ser	95	0.71 ± 0.09	0.78 ± 0.04	0.72 ± 0.05
	125	0.66 ± 0.07^b^	0.71 ± 0.04^b^	0.84 ± 0.11^a^
	65	0.99 ± 0.09	0.91 ± 0.03	0.93 ± 0.04
Thr	95	0.93 ± 0.12^a^	0.97 ± 0.07^a^	0.84 ± 0.05^b^
	125	0.90 ± 0.09^b^	0.93 ± 0.06^ab^	1.03 ± 0.12^a^
	65	0.70 ± 0.05	0.66 ± 0.02	0.67 ± 0.04
Tyr	95	0.61 ± 0.08	0.60 ± 0.17	0.49 ± 0.13
	125	0.72 ± 0.10	0.72 ± 0.09	0.75 ± 0.08
	65	0.92 ± 0.07^b^	1.01 ± 0.04^a^	1.00 ± 0.03^a^
Val	95	0.99 ± 0.11	1.02 ± 0.06	0.92 ± 0.05
	125	1.03 ± 0.10^b^	1.07 ± 0.05^ab^	1.19 ± 0.14^a^
	65	17.96 ± 1.44^b^	19.14 ± 0.59^ab^	19.35 ± 0.81^a^
TAA	95	18.85 ± 2.28^a^	19.36 ± 1.26^a^	16.97 ± 0.84^b^
	125	18.71 ± 1.69^b^	19.59 ± 0.94^b^	21.73 ± 2.49^a^
	65	7.54 ± 0.58	7.97 ± 0.29	7.85 ± 0.43
EAA	95	7.79 ± 0.91^a^	7.87 ± 0.51^a^	6.91 ± 0.32^b^
	125	7.74 ± 0.72^b^	8.06 ± 0.37^b^	9.04 ± 1.16^a^
	65	10.42 ± 0.86^b^	11.17 ± 0.31^a^	11.50 ± 0.45^a^
NEAA	95	11.05 ± 1.39	11.48 ± 0.79	10.06 ± 0.55
	125	10.97 ± 0.99^b^	11.54 ± 0.57^ab^	12.69 ± 1.33^a^
	65	8.65 ± 0.75^b^	9.28 ± 0.27^a^	9.57 ± 0.37^a^
FAA	95	9.19 ± 1.11^ab^	9.66 ± 0.58^a^	8.38 ± 0.44^b^
	125	8.92 ± 0.76^b^	9.47 ± 0.45^b^	10.51 ± 1.19^a^
***Psoas major*** **muscle**
	65	1.19 ± 0.15	1.12 ± 0.06	1.11 ± 0.07
Ala	95	1.25 ± 0.15^a^	1.16 ± 0.08^a^	0.95 ± 0.06^b^
	125	1.13 ± 0.08	1.11 ± 0.06	1.11 ± 0.08
	65	1.31 ± 0.17	1.32 ± 0.10	1.27 ± 0.11
Arg	95	1.33 ± 0.20^a^	1.23 ± 0.10^ab^	1.12 ± 0.04^b^
	125	1.29 ± 0.07	1.26 ± 0.06	1.27 ± 0.07
	65	1.92 ± 0.31^a^	1.76 ± 0.13^ab^	1.61 ± 0.15^b^
Asp	95	1.76 ± 0.23^a^	1.67 ± 0.12^a^	1.48 ± 0.07^b^
	125	1.74 ± 0.13	1.63 ± 0.05	1.62 ± 0.10
	65	2.81 ± 0.44	2.87 ± 0.24	2.83 ± 0.30
Glu	95	2.95 ± 0.46^a^	3.01 ± 0.26^a^	2.43 ± 0.10^b^
	125	2.95 ± 0.18	3.10 ± 0.10	2.90 ± 0.22
	65	0.96 ± 0.13	0.96 ± 0.12	0.88 ± 0.11
Gly	95	0.90 ± 0.15^a^	0.89 ± 0.07^a^	0.77 ± 0.06^b^
	125	0.85 ± 0.05	0.82 ± 0.05	0.83 ± 0.05
	65	0.74 ± 0.10	0.73 ± 0.08	0.84 ± 0.19
His	95	0.86 ± 0.15^a^	0.87 ± 0.16^a^	0.65 ± 0.04^b^
	125	0.74 ± 0.06^c^	0.92 ± 0.06^a^	0.83 ± 0.06^b^
	65	0.93 ± 0.12	0.91 ± 0.08	0.86 ± 0.07
Ile	95	0.97 ± 0.15^a^	0.91 ± 0.07^ab^	0.83 ± 0.03^b^
	125	0.94 ± 0.06	0.91 ± 0.04	0.91 ± 0.05
	65	1.71 ± 0.23	1.61 ± 0.10	1.53 ± 0.15
Leu	95	1.72 ± 0.20^a^	1.66 ± 0.12^a^	1.41 ± 0.06^b^
	125	1.59 ± 0.12	1.56 ± 0.04	1.50 ± 0.07
	65	1.97 ± 0.27	1.78 ± 0.16	1.71 ± 0.15
Lys	95	1.77 ± 0.29	1.73 ± 0.14	1.58 ± 0.07
	125	1.81 ± 0.11	1.75 ± 0.05	1.75 ± 0.11
	65	0.56 ± 0.06	0.55 ± 0.05	0.57 ± 0.06
Met	95	0.59 ± 0.08^a^	0.62 ± 0.06^a^	0.52 ± 0.02^b^
	125	0.55 ± 0.03^b^	0.61 ± 0.01^a^	0.56 ± 0.03^b^
	65	0.81 ± 0.09^b^	0.84 ± 0.08^ab^	0.94 ± 0.12^a^
Phe	95	0.91 ± 0.13^b^	1.03 ± 0.10^a^	0.75 ± 0.05^c^
	125	0.85 ± 0.05^b^	1.01 ± 0.06^a^	0.90 ± 0.04^b^
	65	0.77 ± 0.14^b^	0.92 ± 0.16^a^	1.00 ± 0.12^a^
Pro	95	0.98 ± 0.26	0.94 ± 0.13	0.78 ± 0.14
	125	0.89 ± 0.07^b^	1.01 ± 0.14^a^	1.05 ± 0.08^a^
	65	0.77 ± 0.09	0.76 ± 0.07	0.75 ± 0.11
Ser	95	0.78 ± 0.12^a^	0.76 ± 0.07^a^	0.62 ± 0.03^b^
	125	0.74 ± 0.05	0.74 ± 0.05	0.74 ± 0.06
	65	0.94 ± 0.12	0.90 ± 0.07	0.89 ± 0.08
Thr	95	0.99 ± 0.16^a^	0.91 ± 0.07^ab^	0.79 ± 0.04^b^
	125	0.91 ± 0.06	0.92 ± 0.05	0.93 ± 0.04
	65	0.70 ± 0.09^a^	0.66 ± 0.08^a^	0.44 ± 0.31^b^
Tyr	95	0.66 ± 0.19	0.66 ± 0.05	0.66 ± 0.08
	125	0.70 ± 0.05^a^	0.32 ± 0.26^b^	0.76 ± 0.04^a^
	65	1.00 ± 0.11	0.99 ± 0.08	0.99 ± 0.11
Val	95	1.02 ± 0.16^a^	1.06 ± 0.08^a^	0.89 ± 0.04^b^
	125	1.00 ± 0.06	1.04 ± 0.03	0.99 ± 0.06
	65	19.06 ± 2.37	18.70 ± 1.52	18.24 ± 1.73
TAA	95	19.43 ± 2.78^a^	19.11 ± 1.53^a^	16.22 ± 0.61^b^
	125	18.68 ± 1.09	18.70 ± 0.74	18.66 ± 0.94
	65	7.90 ± 0.97	7.59 ± 0.63	7.49 ± 0.67
EAA	95	7.96 ± 1.11^a^	7.92 ± 0.63^a^	6.77 ± 0.26^b^
	125	7.66 ± 0.47	7.79 ± 0.14	7.55 ± 0.36
	65	11.16 ± 1.43	11.10 ± 0.93	10.74 ± 1.09
NEAA	95	11.47 ± 1.69^a^	11.19 ± 0.93^a^	9.45 ± 0.37^b^
	125	11.03 ± 0.62	10.91 ± 0.68	11.11 ± 0.58
	65	9.36 ± 1.22	9.29 ± 0.75	9.09 ± 0.86
FAA	95	9.60 ± 1.43^a^	9.34 ± 0.73^a^	7.82 ± 0.29^b^
	125	9.21 ± 0.55	9.33 ± 0.43	9.18 ± 0.54

*Data are presented as means ± SE. Values in the same row without a common superscript letter are significantly different (P <0.05)*.

*At 65-, 95-, and 125-d-old, the replicates of the control group were 12, 11, and 8; the sow-betaine group were 6, 7, and 6; and the sow-offspring-betaine group were 7, 7, and 6, respectively*.

1 *includes aspartate plus asparagine;*

2 *includes glutamate plus glutamine. EAA includes Thr, Val, Met, Ile, Leu, Phe, Lys, His, Arg, and Pro; NEAA includes Asp, Ser, Glu, Gly, Ala, and Tyr*.

The BF muscle analysis showed that betaine addition in diets can significantly affect the hydrolyzed amino acid contents. Compared with the control group, betaine addition in the sow and sow-offspring diets increased the contents of Ala, Asp, Glu, Gly, Ile, Lys, Ser, Val, non-essential amino acid (NEAA), and flavor amino acid (FAA), while decreased the content of Pro of 65-d-old pigs (*P* < 0.05). Moreover, betaine addition in the sow-offspring diets increased (*P* < 0.05) the content of total amino acids (TAA) of 65-d-old pigs compared with the pigs in the control group. In the 95-d-old pigs, the contents of Arg, Asp, Gly, Ile, Leu, Lys, Thr, TAA, and EAA were decreased in the sow-offspring-betaine group compared with the other two groups, whereas the content of Glu was increased in the sow-betaine group compared with the other two groups (*P* < 0.05). Moreover, the content of FAA was decreased (*P* < 0.05) in the sow-offspring-betaine group compared with the other two groups. In the 125-d-old pigs, the contents of Ala, Asp, Glu, Ile, Leu, Lys, Ser, TAA, EAA, and FAA were increased in the sow-offspring-betaine group compared with the other two groups, whereas the contents of Gly, Thr, Val, and NEAA were increased in the sow-offspring-betaine group compared with the control group (*P* < 0.05).

In the PM muscle, compared with the control group, betaine addition in the sow-offspring diets decreased (*P* < 0.05) the content of Asp and increased the content of Phe of 65-d-old pigs (*P* < 0.05). In addition, betaine addition in the sow and sow-offspring diets increased the content of Pro compared with the control group, whereas betaine addition in the sow-offspring diets decreased the content of Tyr compared with the other two groups in the 65-d-old pigs (*P* < 0.05). In the 95-d-old pigs, the contents of Ala, Asp, Glu, Gly, His, Leu, Met, Ser, Val, TAA, EAA, NEAA, and FAA were decreased in the sow-offspring-betaine group compared with the other two groups, whereas the contents of Arg, Ile, and Thr were decreased compared with the control group (*P* < 0.05). In the 125-d-old pigs, the contents of Met and Phe were increased in the sow-betaine group, whereas the content of Tyr was decreased compared with the other two groups (*P* < 0.05). Moreover, the contents of His and Pro in the sow-betaine and sow-offspring-betaine groups were increased (*P* < 0.05) compared with the control group in the 125-d-old pigs.

### The mRNA Expression of Genes Related to MyHC Isoform and MRFs in the Muscle

Table 7 presents the level of mRNA gene expressions related to myosin heavy chain (MyHC) isoform and myogenic regulatory factors (MRFs) in LTL, BF, and PM muscles. In the LTL muscle, compared with the control group, the mRNA expression level of *MyHC-IIb* was up-regulated (*P* < 0.05) in the sow-betaine group and the mRNA expression level of myogenic degradation factor 5 (*Myf5*) was up-regulated in the 65-d-old pigs. In the 95-d-old pigs, the mRNA expression levels of *MyHC-I* and Myogenin (*MyoG*) were up-regulated in the sow-offspring-betaine group compared with the control group, whereas the mRNA expression levels of *MyHC-IIx* and *Myf5* up-regulated in the sow-offspring-betaine group compared with the other two groups (*P* < 0.05). Moreover, the mRNA expression level of *MyHC-IIb* in the sow-betaine and sow-offspring-betaine groups was up-regulated (*P* < 0.05) compared with the control group in the 95-d-old pigs. In the 125-d-old pigs, the mRNA expression level of *MyHC-IIx* was up-regulated in the sow-offspring-betaine group compared with the control group.

**Table 7:** Effect of dietary betaine on the expression of genes related to MyHC isoform and MRFs in the muscle of Bama mini-pigs.

**Items**	**d-old**	**Control group**	**Sow-betaine group**	**Sow-offspring-betaine group**
***Longissimus thoracis et lumborum*** **muscle**
	65	1.00 ± 0.25	0.84 ± 0.20	1.25 ± 0.26
*MyHC-I*	95	1.00 ± 0.36^b^	1.93 ± 0.26^ab^	3.31 ± 1.05^a^
	125	1.00 ± 0.19	0.74 ± 0.18	1.60 ± 0.36
	65	1.00 ± 0.20	0.75 ± 0.07	0.99 ± 0.12
*MyHC-IIa*	95	1.00 ± 0.10	1.01 ± 0.08	1.14 ± 0.25
	125	1.00 ± 0.28	0.82 ± 0.06	1.20 ± 0.31
	65	1.00 ± 0.06	1.59 ± 0.33	1.52 ± 0.23
*MyHC-IIx*	95	1.00 ± 0.15^b^	1.25 ± 0.27^b^	2.49 ± 0.15^a^
	125	1.00 ± 0.22^b^	2.21 ± 0.53^ab^	3.81 ± 1.22^a^
	65	1.00 ± 0.11^b^	1.67 ± 0.20^a^	1.51 ± 0.29^ab^
*MyHC-IIb*	95	1.00 ± 0.10^b^	2.03 ± 0.40^a^	2.14 ± 0.26^a^
	125	1.00 ± 0.17	1.26 ± 0.14	1.20 ± 0.28
	65	1.00 ± 0.09	1.00 ± 0.22	0.65 ± 0.13
*MSTN*	95	1.00 ± 0.15	0.89 ± 0.18	1.62 ± 0.45
	125	1.00 ± 0.15	1.06 ± 0.13	1.28 ± 0.19
	65	1.00 ± 0.17^b^	0.81 ± 0.11^b^	1.94 ± 0.49^a^
*Myf5*	95	1.00 ± 0.08^b^	1.15 ± 0.23^b^	2.81 ± 0.67^a^
	125	1.00 ± 0.21	0.60 ± 0.07	0.90 ± 0.26
	65	1.00 ± 0.13	1.12 ± 0.08	1.02 ± 0.14
*MyoG*	95	1.00 ± 0.21^b^	1.64 ± 0.29^ab^	2.27 ± 0.39^a^
	125	1.00 ± 0.12	0.83 ± 0.09	0.66 ± 0.10
***Biceps femoris*** **muscle**
	65	1.00 ± 0.50	1.64 ± 0.68	1.46 ± 1.15
*MyHC-I*	95	1.00 ± 0.72	0.93 ± 0.34	1.61 ± 0.90
	125	0.83 ± 0.17	1.36 ± 0.51	1.14 ± 0.37
	65	1.00 ± 1.91	0.71 ± 0.53	0.55 ± 0.49
*MyHC-IIa*	95	1.01 ± 0.46	0.70 ± 0.18	1.27 ± 0.50
	125	1.13 ± 0.48	0.93 ± 0.54	1.33 ± 0.51
	65	1.01 ± 0.41	2.04 ± 0.85	1.67 ± 1.61
*MyHC-IIx*	95	1.00 ± 0.78	0.61 ± 0.10	0.89 ± 0.27
	125	1.12 ± 0.63	0.92 ± 0.72	1.06 ± 0.35
	65	1.00 ± 0.43	1.32 ± 0.24	26.54 ± 58.19
*MyHC-IIb*	95	1.00 ± 0.45^ab^	0.67 ± 0.31^b^	2.00 ± 1.74^a^
	125	0.64 ± 0.45^b^	1.80 ± 1.06^a^	0.55 ± 0.24^b^
	65	1.00 ± 1.08	0.68 ± 0.44	2.23 ± 3.48
*MSTN*	95	1.00 ± 0.73	0.60 ± 0.30	1.03 ± 0.41
	125	1.00 ± 0.42	1.34 ± 0.82	1.05 ± 0.33
	65	1.00 ± 0.36	1.62 ± 0.92	4.92 ± 6.02
*Myf5*	95	1.00 ± 1.18	0.41 ± 0.23	0.85 ± 0.73
	125	1.01 ± 0.95	1.49 ± 1.81	0.51 ± 0.25
	65	1.00 ± 1.99	0.55 ± 0.48	0.88 ± 1.21
*MyoG*	95	1.00 ± 0.69	0.67 ± 0.21	0.90 ± 0.25
	125	0.95 ± 0.36	1.03 ± 0.31	0.81 ± 0.23
***Psoas major*** **muscle**
	65	1.00 ± 0.43^b^	3.64 ± 2.20^a^	2.58 ± 1.43^a^
*MyHC-I*	95	1.00 ± 0.44	1.04 ± 0.47	0.69 ± 0.22
	125	1.00 ± 1.12	0.93 ± 0.64	0.51 ± 0.25
	65	1.00 ± 0.59^b^	4.97 ± 4.54^a^	2.47 ± 2.19^ab^
*MyHC-IIa*	95	1.00 ± 0.37	0.89 ± 0.35	0.85 ± 0.41
	125	1.00 ± 0.23	1.80 ± 1.43	1.29 ± 1.60
	65	1.00 ± 0.65	0.67 ± 0.47	0.50 ± 0.42
*MyHC-IIx*	95	1.00 ± 0.80	1.52 ± 1.05	1.37 ± 0.52
	125	1.00 ± 0.72	2.00 ± 2.29	1.02 ± 0.88
	65	1.00 ± 0.86^b^	3.62 ± 2.88^a^	3.22 ± 1.94^a^
*MyHC-IIb*	95	1.00 ± 0.97	0.99 ± 0.48	0.79 ± 0.30
	125	1.00 ± 0.61	0.87 ± 0.44	0.78 ± 0.24
	65	1.00 ± 0.51	7.21 ± 11.44	1.54 ± 0.79
*MSTN*	95	1.00 ± 0.38	2.19 ± 2.65	2.23 ± 1.31
	125	1.00 ± 0.48	0.98 ± 0.51	1.15 ± 0.51
	65	1.00 ± 0.39	1.67 ± 0.70	1.20 ± 0.86
*Myf5*	95	1.00 ± 0.66	1.21 ± 1.35	1.68 ± 1.02
	125	1.00 ± 0.88	1.05 ± 1.27	0.91 ± 0.58
	65	1.00 ± 0.54^b^	6.25 ± 6.75^a^	1.88 ± 0.64^b^
*MyoG*	95	1.00 ± 0.42	0.90 ± 0.37	0.63 ± 0.35
	125	1.00 ± 0.20^a^	0.95 ± 0.30^a^	0.60 ± 0.20^b^

*Data are presented as means ± SE. Values in the same row without a common superscript letter are significantly different (P < 0.05)*.

*At 65-, 95-, and 125-d-old, the replicates of the control group were 12, 11, and 8; the sow-betaine group were 6, 7, and 6; and the sow-offspring-betaine group were 7, 7, and 6, respectively*.

*MyHC-IIa, Myosin heavy chain-IIa; MyHC-IIb, Myosin heavy chain-IIb; Myf5, Myogenic degradation factor; MyHC-I, Myosin heavy chain-I; MyHC-IIx, Myosin heavy chain-IIx; MyoG, Myogenin; MSTN, Myostation*.

In the BF muscle, in the 95-d-old pigs, the mRNA expression level of *MyHC-IIb* was up-regulated (*P* < 0.05) in the sow-offspring-betaine group compared with the sow-betaine group. Moreover, in the 125-d-old pigs, the mRNA expression level of *MyHC-IIb* was up-regulated (*P* < 0.05) in the sow-betaine group compared with the other two groups.

In the PM muscle, in the 65-d-old pigs, the mRNA expression levels of *MyHC-I* and *MyHC-IIb* were up-regulated (*P* < 0.05) in the sow-betaine and sow-offspring-betaine groups compared with the control group, and the level of *MyHC-IIa* was up-regulated in the sow-betaine group. The mRNA level of *Myf5* of 65-d-old pigs in the sow-betaine group was up-regulated (*P* < 0.05) compared with the 65-d-old pigs in the other two groups. Moreover, in the 125-d-old pigs, the mRNA expression level of *MyoG* was down-regulated (*P* < 0.05) in the sow-offspring-betaine group compared with the other two groups. However, betaine supplementation had no impacts (*P* > 0.05) on the mRNA expression levels of PM muscle in the 95-d-old pigs.

## Discussion

As an antibiotic substitute in livestock production, dietary betaine has gained more attraction because of its growth-promoting effect. For example, betaine added to the diet can significantly increase the growth hormone (GH) level and total protein content in the blood to improve the utilization of the protein and the growth of finishing pigs (29). Betaine addition in the broiler chicken's diet could improve antioxidant defenses and decrease the breast muscles' lipid peroxidation (30). Furthermore, a recent study suggested that betaine (500, 1,000, and 2,000 mg kg^−1^) can improve the nitrogen retention, growth performance, digestive function, carcass characteristics, and meat quality of broilers under heat stress (31). Similarly, we found that the betaine addition in the sow and offspring diets significantly changes the carcass traits, meat quality, and nitrogen metabolism of Bama-mini-pigs.

Carcass traits and meat quality can intuitively reflect the digestion, absorption, and deposition of the nutrients in the body. Dietary compositions can directly or indirectly affect the metabolism of nutrients in the body and thereby change the meat quality and carcass traits. For example, Cheng et al. (32) found that low amino acid content in diets can effectively improve the meat quality, such as muscle tender and fat content. In the present study, the betaine addition to the sow and offspring diets could effectively increase the carcass weight, carcass yield, backfat thickness, and loin-eye area of 65-, 95-, and 125-d-old Bama mini-pigs. In light of the previous findings by Zhan et al. (33) in broiler chickens, dietary betaine could act as a methyl donor and increase the utilization of amino acids, thereby promote protein synthesis, increase the carcass lean meat rate, and improve carcass quality (34). Meat quality is generally measured by two parts, the sensory and nutrients indexes. The present study found that betaine addition to sows and offspring diets could significantly increase the shear force, brightness, and crude protein content of 125-d-old pigs, while betaine addition to sows could decrease the marbling score of LTL muscles of 65- and 95-d-old pigs, respectively. These findings are inconsistent with the results of Lawrence et al. (35). The possible explanation of the discrepancy may be caused by the protein deposition, which is faster than the lipid in the early life of pigs.

Protein is the most important and nutritious component of meat, and consumers are concerned most about it (36). The composition and content of amino acid shape the nutritional value and flavor of the meat. Therefore, we evaluated the amino acid content in the plasma and muscle to investigate how dietary betaine affects the metabolism of amino acid. The results showed that the betaine addition in the sow-offspring diets significantly improved the contents of various free amino acids in the plasma, such as Asp, Met, Ser, and Glu at different stages. Meanwhile, Glu plays an important role in intestinal, cardiovascular, and neurodevelopment, and Met and Ser are the important FAAs (37). Because of the methyl donor properties, dietary betaine addition can enhance the methionine, and produce a variety of secondary metabolites to promote the synthesis of other EAAs, such as glycine and serine, eventually improves muscle flavor and meat quality (38). In the present study, the amino acids contents in different muscles (LTL, BF, and PM) and different stages (65-, 95-, and 125-d-old) of pigs were varied, several EAAs like Met, His, Ile, Lys, and Phe were significantly increased in the LTL muscle, whereas those EAAs were decreased in the BF and PM muscles. The reduction of these EAAs in the PM muscle may be caused by the different enzyme activities in different tissues, and further studies are necessary to reveal the specific mechanism.

The plasma biochemical parameters are the important indicator for judging the body's health, which can reflect the nutritional metabolism and the functions of various tissues and organs. Total protein content can reflect protein synthesis and metabolism to a certain extent and positively correlated with muscle rate (39). The plasma AST is an important transaminase in the process of amino acid metabolism; its activity is closely related to the body's liver physiological functions and the intensity of amino acid metabolism (40). The present study showed that the betaine addition in sow diets increased the plasma TP content in the 65-d-old pigs, while betaine addition in sow and offspring diets increased the plasma AST content. These findings indicated that dietary betaine could promote protein synthesis, including the low-density lipoproteins in the liver.

Skeletal muscle is the main meat-producing tissue of pigs. The number and diameter of muscle fibers are important factors in muscle formation (41). Moreover, the growth and development of muscle fibers also play an important role in meat quality. The type of muscle fiber has been determined during the fetal period. The muscle fiber types of pigs during the fetal period are mainly *MyHC-I* and *MyHC-IIa*, and the muscle fiber types of newborn piglets are mainly *MyHC-I, MyHC-IIa*, and *MyHC-IIx*. While *MyHC-IIb* is less, and *MyHC-IIb* muscle fibers will continue increasing with muscles developing (42, 43). In the present study, betaine addition in sow-offspring diets up-regulated the mRNA expression levels of *MyHC-I, MyHC-IIx, MyHC-IIb*, and *MyoG* genes of the muscles at different stages. The results were consistent with the previous findings by Zhuo et al. (44) who studied the effects of methyl donor supplementation in the sow diet on piglets' muscle growth. In addition, the present study also found that betaine addition to sows and their offspring' diets significantly up-regulated the *Myf5* and *MyoG* gene expressions in the LTL muscle. The *Myf5* and *MyoG* belong to the MRF genes family. The expression of MRF has great significance to the growth, development, and differentiation of skeletal muscle (45). Therefore, the present study indicated that dietary betaine can regulate the expression level of muscle-derived regulatory factors and genes related to muscle fiber types to influence the growth and development of skeletal muscle.

## Conclusions

In summary, betaine addition in the sow and piglets' diets could increase the carcass weight, carcass yield, lean meat rate, meat color, shear force, and crude protein content of muscles at different stages. Furthermore, betaine addition in sow-offspring diets upregulated the expression level of *MyHC-I, MyHC-IIx, MyHC-IIb*, and *MyoG*, while dietary betaine addition in the sow and sow-offspring diets improved the contents of muscle amino acids. However, betaine addition in the sow-offspring diet had more distinct effects than the betaine addition in sow diet. Therefore, these findings provide the reference for research into dietary betaine addition in the sow and piglets diets to improve the carcass traits and meat quality of Bama mini-pigs.

## Data Availability Statement

The original contributions presented in the study are included in the article/Supplementary Material, further inquiries can be directed to the corresponding author/s.

## Ethics Statement

All procedures were approved by the Animal Care and Use Committee of the Institute of Subtropical Agriculture, Chinese Academy of Sciences (ISA-2018-071).

## Author Contributions

YC, MS, QZ, and MA performed sampling and nutrient measurements, analyzed data, interpreted the results, and drafted the manuscript. YC, MS, and QG conducted animal feeding and sampling. XK contributed to experimental concepts and design, provided scientific direction, and together with MA finalized the manuscript. All authors have read and approved the final manuscript.

## Conflict of Interest

The authors declare that the research was conducted in the absence of any commercial or financial relationships that could be construed as a potential conflict of interest.

## Publisher's Note

All claims expressed in this article are solely those of the authors and do not necessarily represent those of their affiliated organizations, or those of the publisher, the editors and the reviewers. Any product that may be evaluated in this article, or claim that may be made by its manufacturer, is not guaranteed or endorsed by the publisher.

## References

[1] Godfray HCJ Aveyard P Garnett T Hall JW Key TJ Lorimer J. Meat consumption, health, and the environment. Science. (2018) 361:eaam5324. 10.1126/science.aam5324 30026199

[2] Mozes S Bujnakova D Sefcikova Z Kmet V. Developmental changes of gut microflora and enzyme activity in rat pups exposed to fat-rich diet. Obesity. (2008) 16:2610–5. 10.1038/oby.2008.435 18927555

[3] Sugiharto S. Role of nutraceuticals in gut health and growth performance of poultry. J Saudi Soc Agric Sci. (2016) 15:99–111. 10.1016/j.jssas.2014.06.001

[4] Huang Q Xu Z Han X Li W. Effect of dietary betaine supplementation on lipogenic enzyme activities and fatty acid synthase mRNA expression in finishing pigs. Anim Feed Sci Technol. (2008) 140:365–75. 10.1016/j.anifeedsci.2007.03.007

[5] Amerah AM Ravindran V. Effect of coccidia challenge and natural betaine supplementation on performance, nutrient utilization, and intestinal lesion scores of broiler chickens fed suboptimal level of dietary methionine. Poult Sci. (2015) 94:673–80. 10.3382/ps/pev022 25691757 PMC4990982

[6] Park SO Kim WK. Effects of betaine on biological functions in meat-type ducks exposed to heat stress. Poult Sci. (2016) 96:1212–8. 10.3382/ps/pew359 27702920

[7] Eklund M Bauer E Wamatu J Mosenthin R. Potential nutritional and physiological functions of betaine in livestock. Nutr Res Rev. (2005) 18:31–48. 10.1079/NRR200493 19079893

[8] Hu L Fei H Chen L Peng X Chen DW Wu D. High nutrient intake during the early postnatal period accelerates skeletal muscle fiber growth and maturity in intrauterine growth-restricted pigs. Genes Nutr. (2018) 13:23. 10.1186/s12263-018-0612-8 30065792 PMC6062929

[9] Oksbjerg N Nissen PM Therkildsen M Møller HS Larsen LB Andersen M. Meat science and muscle biology symposium: *in utero* nutrition related to fetal development, postnatal performance, and meat quality of pork. J Anim Sci. (2013) 91:1443–53. 10.2527/jas.2012-5849 23296813

[10] Zou T He D Yu B Yu J Mao X Zheng P. Moderately increased maternal dietary energy intake delays foetal skeletal muscle differentiation and maturity in pigs. Eur J Nutr. (2016) 55:1777–87. 10.1007/s00394-015-0996-9 26179476

[11] Hines EA Coffey JD Starkey CW Chung TK Starkey JD. Improvement of maternal vitamin D status with 25-hydroxycholecalciferol positively impacts porcine fetal skeletal muscle development and myoblast activity. J Anim Sci. (2013) 91:4116–22. 10.2527/jas.2013-6565 23893976

[12] Jiang YZ Zhu L Li XW Si T. Evaluation of the Chinese indigenous pig breed dahe and crossbred dawu for growth and carcass characteristics, organ weight, meat quality and intramuscular fatty acid and amino acid composition. Animal. (2011) 5:1485–92. 10.1017/S1751731111000425 22440295

[13] Yang L Bian G Su Y Zhu W. Comparison of faecal microbial community of Lantang, Bama, Erhualian, Meixshan, Duroc, Landrace, and Yorkshire sows. Asian-Australas J Anim Sci. (2014) 27:898–906. 10.5713/ajas.2013.13621 25050029 PMC4093183

[14] Yang J Dai L Yu Q Yang Q. Histological and anatomical structure of the nasal cavity of Bama minipigs. PLoS ONE. (2017) 12:e0173902. 10.1371/journal.pone.0173902 28339502 PMC5365122

[15] Ma C Gao Q Zhang W Azad MAK Kong X. Alterations in the blood parameters and fecal microbiota and metabolites during pregnant and lactating stages in Bama mini pigs as a model. Mediat Inflamm. (2020) 2020:8829072. 10.1155/2020/8829072 33162832 PMC7607286

[16] Wang K Kong X Azad MAK Zhu Q Xiong L Zheng Y. Maternal probiotic or synbiotic supplementation modulates jejunal and colonic antioxidant capacity, mitochondrial function, and microbial abundance in Bama mini-piglets. Oxid Med Cell Longev. (2021) 2021:6618874. 10.1155/2021/6618874 34035877 PMC8116152

[17] Wang AD Lan GQ Guo YF. Genetic breeding of guangxi Bama mini-pig. Lab Anim Sci. (2010) 27:60–63.

[18] Zhu X Wei Y Zhan Q Yan A Tang D. CRISPR/Cas9-mediated biallelic knockout of IRX3 reduces the production and survival of somatic cell-cloned Bama minipigs. Animals. (2020) 10:501. 10.3390/ani10030501 32192102 PMC7142520

[19] Gao Q Ma C Kong X Yin F Han Q Yin Y. Effects of dietary betaine supplementation on reproductive performance, colostrum composition and plasma metabolite and reproductive hormone contents of Bama mini-pigs. Chin J Anim Nutr. (2020) 32:646–53. 10.3969/j.issn.1006-267x.2020.02.020

[20] Gao Q Ma C Song M Wang Z Yin F Hang Q. Effects of betaine supplementation in sow diets on blood indexes of suckling Bama mini-pigs. Chin J Anim Nutr. (2020) 32:925–31. 10.3969/j.issn.1006-267x.2020.02.048

[21] Azad MAK Gao Q Ma C Wang K Kong X. Betaine hydrochloride addition in Bama mini-pig's diets during gestation and lactation enhances immunity and alters intestine microbiota of suckling piglets. J Sci Food Agric. (2021). 10.1002/jsfa.11389. [Epub ahead of print]. 34151432

[22] China Agriculture Press. Ministry of Agriculture of the People's Republic of China Feeding Standard of Swine (GB, NY/T 65-2004). Beijing: China Agriculture Press (2004).

[23] National Research Council. Nutrient Requirements of Swine: Eleventh Revised Edition. Washington, DC: The National Academies Press (2012).

[24] Hu C Jiang Q Zhang T Yin Y Li F Su JY. Dietary supplementation with arginine and glutamic acid enhances key lipogenic gene expression in growing pigs. J Anim Sci. (2017) 95:5507–15. 10.2527/jas2017.1703 29293787 PMC6292291

[25] Christensen LB. Drip loss sampling in porcine *M. longissimus dorsi*. Meat Sci. (2003) 63:469–77. 10.1016/S0309-1740(02)00106-7 22062516

[26] Liu Y Li F Kong X Li Y Duan Y Blachier F. Signaling pathways related to protein synthesis and amino acid concentration in pig skeletal muscles depend on the dietary protein level, genotype and developmental stages. PLoS ONE. (2015) 10:e0138277. 10.1371/journal.pone.0138277 26394157 PMC4578863

[27] Dong L Zhong X Zhang L Kong L Kong Y Kou T. Impaired intestinal mucosal immunity is associated with the imbalance of T lymphocyte sub-populations in intrauterine growth-restricted neonatal piglets. Immunobiology. (2015) 220:775–81. 10.1016/j.imbio.2014.12.017 25791524

[28] Livak KJ Schmittgen TD. Analysis of relative gene expression data using real-time quantitative PCR and the 2^−Δ*ΔCT*^ method. Methods. (2001) 25:402–8. 10.1006/meth.2001.1262 11846609

[29] Huang Q Xu Z Han X Li W. Effect of betaine on growth hormone pulsatile secretion and serum metabolites in finishing pigs. J Anim Physiol Anim Nutr. (2007) 91:85–90. 10.1111/j.1439-0396.2006.00644.x 17355337

[30] Alirezaei M Gheisari HR Ranjbar VR Hajibemani A. Betaine: a promising antioxidant agent for enhancement of broiler meat quality. Br Poult Sci. (2012) 53:699–707. 10.1080/00071668.2012.728283 23281766

[31] Liu W Yuan Y Sun C Balasubramanian B Zhao Z An L. Effects of dietary betaine on growth performance, digestive function, carcass traits, and meat quality in indigenous yellow-feathered broilers under long-term heat stress. Animals. (2019) 9:506. 10.3390/ani9080506 31370305 PMC6720770

[32] Cheng C Liu Z Zhou Y Wei H Zhang X Xia M. Effect of oregano essential oil supplementation to a reduced-protein, amino acid-supplemented diet on meat quality, fatty acid composition, and oxidative stability of *Longissimus thoracis* muscle in growing-finishing pigs. Meat Sci. (2017) 133:103–9. 10.1016/j.meatsci.2017.06.011 28666108

[33] Zhan XA Li JX Xu ZR Zhao RQ. Effects of methionine and betaine supplementation on growth performance, carcase composition and metabolism of lipids in male broilers. Br Poult Sci. (2006) 47:576–80. 10.1080/00071660600963438 17050102

[34] Yang Z Wang ZY Yang HM Zhao FZ Kong LL. Response of growing goslings to dietary supplementation with methionine and betaine. Br Poult Sci. (2016) 57:833–41. 10.1080/00071668.2016.1230663 27717289

[35] Lawrence BV Schinckel A Adeola O Cera K. Impact of betaine on pig finishing performance and carcass composition. J Anim Sci. (2002) 80:475–82. 10.2527/2002.802475x 11881932

[36] Pereira PMDCC Vicente AFDRB. Meat nutritional composition and nutritive role in the human diet. Meat Sci. (2013) 93:586–92. 10.1016/j.meatsci.2012.09.018 23273468

[37] Lorenzo JM Franco D. Fat effect on physico-chemical, microbial and textural changes through the manufactured of dry-cured foal sausage lipolysis, proteolysis and sensory properties. Meat Sci. (2012) 92:704–14. 10.1016/j.meatsci.2012.06.026 22795774

[38] Craig SAS. Betaine in human nutrition. Am J Clin Nutr. (2004) 80:539–49. 10.1093/ajcn/80.3.539 15321791

[39] Kapelański W Grajewska S Bocian M Dybała J Jankowiak H Wiśniewska J. Changes in blood biochemical indicators during fattening of the high-lean pigs. Anim Sci Pap Rep. (2004) 4:443–9.

[40] Piotrowska A Burlikowska K Szymeczko R. Changes in blood chemistry in broiler chickens during the fattening period. Folia Biol. (2011) 59:183–7. 10.3409/fb59_3-4.183-187 22195474

[41] Gao P Cheng Z Li M Zhang N Le B Zhang W. Selection of candidate genes affecting meat quality and preliminary exploration of related molecular mechanisms in the mashen pig. Asian-Australas J Anim Sci. (2019) 32:1084–94. 10.5713/ajas.18.0718 31010998 PMC6599955

[42] Hua N Takahashi H Yee GM Kitajima Y Katagiri S Kojima M. Influence of muscle fiber type composition on early fat accumulation under high-fat diet challenge. PLoS ONE. (2017) 12:e0182430. 10.1371/journal.pone.0182430 28763507 PMC5538743

[43] Katsumata M Yamaguchi T Ishida A Ashihara A. Changes in muscle fiber type and expression of mRNA of myosin heavy chain isoforms in porcine muscle during pre- and postnatal development. Anim Sci J. (2017) 88:364–71. 10.1111/asj.12641 27230088

[44] Zhuo Y Wang J Liu H Mou D Adebowale T Che L. Effects of maternal methyl donor on the pork characteristics of offspring pigs with prenatal exposure to bisphenol A. Animal. (2017) 12:1306–15. 10.1017/S175173111700252X 29065941

[45] Harding RL Halevy O Yahav S Velleman SG. The effect of temperature on proliferation and differentiation of chicken skeletal muscle satellite cells isolated from different muscle types. Physiol Rep. (2016) 4:e12770. 10.14814/phy2.12770 27125667 PMC4848725

