# Supplementary material for: Dietary Betaine Addition Alters Carcass Traits, Meat Quality, and Nitrogen Metabolism of Bama Mini-Pigs
Source: Front Nutr. 2021 Aug 27;8:728477. doi: 10.3389/fnut.2021.728477 (PMC8429818; doi:10.3389/fnut.2021.728477)
Supplement: Supplementary file 1 [file Data_Sheet_1.PDF]

Supplementary Material

Table S1. Composition and nutrient levels of the basal diets for sows (air-dry basis; %)

Items	Pregnant sows' diet	Lactating sows' diet
Ingredients		
Corn	37.50	66.00
Soybean meal	9.50	25.00
Wheat bran	14.00	5.00
Barley	25.00	0.00
Soybean hull	10.00	0.00
Pregnant sows' premix[1)]	4.00	0.00
Lactating sows' premix[2)]	0.00	4.00
Total	100.00	100.00
Nutrient levels[3)]		
Digestive Energy (MJ/Kg)	12.55	13.87
Crude Protein	12.82	16.30
Lys	0.48	0.75
Met+Cys	0.43	0.51
Thr	0.37	0.53
Trp	0.13	0.17
Ca	0.62	0.65
Total P	0.47	0.50

Note: [1)]Pregnant sows' premix provided the following per kg of the diet: Ca, 2.32 g; Na, 6.75 g; Cu, 20.48 mg; Fe, 118.6 mg; Zn, 87.15 mg; Mn, 32.54 mg; Mg, 0.1 g; I, 39 mg; Se, 16.44 mg; Co, 7.26 mg; VA, 10 000 IU; VE, 20 mg; VK_3, 2.4 mg; VB_1, 1.6 mg; VB_2, 6 mg; VB_6, 1.6 mg; VB_{12}, 0.024 mg; folic acid, 1.2 mg; nicotinamide, 20 mg; pantothenic acid, 12 mg; biotin, 0.12 mg; ferrous glycinate, 100 mg; choline chloride, 1 g; phytase, 200 mg; flavoring agent, 80 mg; and limestone, 12 g.

[2)] Lactating sows' premix provided the following per kg of the diet: Ca, 2.32 g; Na, 6.75 g; Cu, 20.48 mg; Fe, 118.6 mg; Zn, 87.15 mg; Mn, 32.54 mg; I, 39 mg; Se, 16.44 mg; Co, 7.26 mg; VA, 10 000 IU; VD_3, 1 800 IU; VE, 20 mg; VK_3, 2.4 mg; VB_1, 1.6 mg; VB_2, 6 mg; VB_6, 1.6 mg; VB_{12}, 0.024 mg; folic acid, 1.2 mg; nicotinamide, 20 mg; pantothenic acid, 12 mg; biotin, 0.12 mg; ferrous glycinate, 100 mg; choline chloride, 1 g; phytase, 200 mg; flavoring agent, 80 mg; and limestone, 12 g.

[3)] Nutrient levels were calculated values.

Table S2. Composition and nutrient levels of basal diets for weaned pigs (air-dry basis; %)

Items	Pre-nursery diet (35-95 day-old)	Post nursery diet (96-125 day-old)
Ingredients		
Corn	54.92	58.00
Soybean meal	22.00	18.35
Wheat bran	10.13	11.35
Rice bran	8.95	8.30
Premix[1)]	4.00	4.00
Total	100.00	100.00
Nutrient levels[2)]		
Digestive Energy (MJ/kg)	13.50	13.42
Crude Protein	16.13	14.90
Lys	1.40	1.30
Met + Cys	0.69	0.66
Thr	0.78	0.74
Ca	0.45	0.44
Total P	0.49	0.49

Note: [1)] Premix provided the following per kilogram of diets: enzymic preparation 1.2 g, VA 26 000 IU, VD_3 10 000 IU, VE 70 IU, VK_3 10 mg, VB_1 10 mg, VB_2 25 mg, VB_6 10 mg, VB_{12} 0.075 mg, biotin 0.4 mg, folic acid 5 mg, nicotinamide 100 mg, pantothenic 50 mg, choline 1 600 mg, flavoring agent 500 mg, edulcorant 300 mg, acidulating agent 5 g, Cu 230 mg, Mn 97 mg, Zn 218 mg, Fe 165 mg, I 0.3 mg, Se 0.3 mg, Co 0.4 mg, glucose 2.1 g, antioxidants 0.4 g, anti-mildew agent 1 g, Ca 3.42 g, and P 1.155 g.

[2)] Nutrient levels were calculated values.

Table S3. Primer sequences of the target genes

Target Genes	Accession No.		Primer Sequences (5′-3′)
β-actin	XM_021086047.1	F:	GGCACCACACCTTCTACAACGAG
		R:	TCATCTTCTCACGGTTGGCTTTGG
MSTN	NM_214435.2	F:	GCACCAAGCAAACCCCAGAGG
		R:	AGCACCCACAGCGATCTACTACC
MYF5	NM_001278775.1	F:	GGATCAGCAACTCCGAGCAACC
		R:	GCACATGGTAGATGAGCCTGGAAC
MyHC-I	NM_213855.2	F:	CTGTCCAAGTTCCGCAAGGT
		R:	CTTTGTTGCGCCCTCAGGAT
MyHC-IIa	NM_214136.1	F:	ACCTACAGCACCGTCTGGATGAG
		R:	TGCTCACTCTCAACCTCTCCTTCC
MyHC-IIb	NM_001123141.1	F:	GGAGCTGAGATGCCTCTGTC
		R:	TCATGGCTGCGGGTTATTGA
MyHC-IIx	NM_001104951.2	F:	TGAGGAAGACCGCAAGAACA
		R:	GGTCACTTTTGAGCATTTGGATG
MYOG	NM_001012406.1	F:	AAACTACCTGCCCGTCCACCTC
		R:	GGTCCCCAGCCCCTTATCTTCC

Note: *MyHC-IIa*, Myosin heavy chain-IIa; *MyHC-IIb*, Myosin heavy chain-IIb; *Myf5*, Myogenic degradation factor; *MyHC-I*, Myosin heavy chain-I; *MyHC-IIx*, Myosin heavy chain-IIx; *MyoG*, Myogenin; *MSTN*, Myostation.
